# Human-Robotic Variable-Stiffness Grasps of Small-Fruit Containers Are Successful Even Under Severely Impaired Sensory Feedback

**DOI:** 10.3389/fnbot.2018.00070

**Published:** 2018-10-31

**Authors:** Mark Haas, Werner Friedl, Georg Stillfried, Hannes Höppner

**Affiliations:** ^1^German Aerospace Center DLR e.V., Institute of Robotics and Mechatronics, Wessling, Germany; ^2^Faculty of Electrical Engineering, University of Applied Sciences Kempten, Kempten, Germany; ^3^Agile Robots AG, Wessling, Germany

**Keywords:** soft manipulation, variable impedance, variable stiffness, grip stiffness, surface electromyography (sEMG), force feedback (FF), visual feedback (VF), environmental constraints

## Abstract

Application areas of robotic grasping extend to delicate objects like groceries. The intrinsic elasticity offered by variable-stiffness actuators (VSA) appears to be promising in terms of being able to adapt to the object shape, to withstand collisions with the environment during the grasp acquisition, and to resist the weight applied to the fingers by a lifted object during the actual grasp. It is hypothesized that these properties are particularly useful in the absence of high-quality sensory feedback, which would otherwise be able to guide the shape adaptation and collision avoidance, and that in this case, VSA hands perform better than hands with fixed stiffness. This hypothesis is tested in an experiment where small-fruit containers are picked and placed using a newly developed variable-stiffness robotic hand. The grasp performance is measured under different sensory feedback conditions: full or impaired visual feedback, full or impaired force feedback. The hand is switched between a variable-stiffness mode and two fixed-stiffness modes. Strategies for modulating the stiffness and exploiting environmental constraints are observed from human operators that control the robotic hand. The results show consistently successful grasps under all stiffness and feedback conditions. However, the performance is affected by the amount of available visual feedback. Different stiffness modes turn out to be beneficial in different feedback conditions and with respect to different performance criteria, but a general advantage of VSA over fixed stiffness cannot be shown for the present task. Guidance of the fingers along cracks and gaps is observed, which may inspire the programming of autonomously grasping robots.

## 1. Introduction

Online grocery sales are an intensively growing field with growth rates of more than 25% year-over-year in the USA (Springer, 2017). Some online supermarkets have their own warehouses, where the groceries are packed into delivery totes before they can be distributed to the customers. The consignment of food is partly done in cold storage rooms to preserve their freshness, which makes the working environment un-alluring for human workers. Letting robots do the task might be one solution. However, automated food grasping is still ambitious.

Robotic grasping and manipulation of grocery items in an online supermarket warehouse poses the challenge of having to deal with thousands of items of different size, shape, or rigidity (Burgess, 2017). Furthermore, some of the items are very delicate but should not be damaged to avoid complaints by customers. To save some of the cost and complexity of perception systems, it is desirable that the robotic systems are able to fulfill their tasks even when high-fidelity sensory feedback is lacking or even missing.

Solutions to these problems are investigated within the European project *Soft-bodied intelligence for Manipulation* (SOMA). It focuses on the development of manipulation systems with simple, robust, and efficient robotic hands with embodied compliance, which both endure and require the exploitation of environmental constraints like surfaces and edges for guiding their motion.

Concepts for embodied compliance include series-elastic actuators (SEA, Pratt and Williamson, 1995), variable-stiffness actuators (VSA, Vanderborght et al., 2013, Wolf et al., 2015) and other concepts such as pneumatics in combination with continuously deformable material (Deimel and Brock, 2015). While SEA systems and the pneumatic system by Deimel and Brock exhibit fixed relationships between their applied force and grip stiffness, VSA systems are equipped with additional motors to change their stiffness characteristics.

Robotic systems with embodied compliance yield various advantages over mechanically rigid systems with sensor-based actively controlled compliance: shock absorption and energy storage for cyclic or explosive movements. Active compliance control also introduces a controller-dependent delay which can lead to potential damage because of peak loads during impacts (Haddadin et al., 2007) whereas the inherent elasticity of SEA and VSA can be used without delay. Furthermore, embodied mechanical compliance might even be realized at a lower cost than actively controlled compliance because of the eliminated need for stiff torque sensors, which are expensive.

Whenever lack of sensory feedback disables a closed-loop control of forces and positions, compliance, i.e., low stiffness, remains as a possibility to guide the robotic hand along environmental constraints and to shape the grip around objects, increasing possible contact points which in return boost the chances of a successful and efficient grasp (Eppner et al., 2015). On the other hand, high stiffness is still required for withstanding the weight of an object during lifting and yields lower positional errors. Since VSA are able to provide both low and high stiffness, we hypothesize that in the absence of high-fidelity sensory feedback, VSA systems are particularly useful and perform better than SEA systems with a fixed stiffness. To test this, we design a manipulation task where visual and force feedback can be switched on and off.

In this paper, a VSA robotic hand prototype—the *DLR Wearable Hand to Investigate Stiffness in Grasping* (WHISG)—is presented and its performance in a particular grocery manipulation task—picking small-fruit containers from storage boxes—is examined.

The WHISG bases its VSA technology on the one of the DLR Hand Arm System (Friedl et al., 2011) and aims to provide decent force and speed at low cost and light weight.

The present study aims at testing the hypothesis that variable stiffness helps better to compensate lack of sensory feedback than fixed stiffness, i.e., that VSA systems suffer less performance deficits than SEA system when sensory feedback is reduced. Furthermore, it aims at answering the questions how in VSA systems stiffness should be modulated and how environmental constraints can guide the robotic hand to a successful grasp position.

For the comparison of VSA and SEA, the manipulation task is attempted with three different stiffness modes of the WHISG hand: variable stiffness, fixed low stiffness, and fixed high stiffness. Please note: even if the underlying mechanism is always a VSA, the hand is exactly used as SEA hand for the two fixed stiffness modes.

To initially save programming effort and learn from human manipulation strategies, instead of being positioned by a robotic arm and controlled by a computer, the robotic hand is positioned and controlled by human operators. For the positioning, the robotic hand is physically attached to the operator's forearm. For the control, the desired robotic hand opening angle is electronically mapped from the operator's hand opening angle. Additionally, in the variable-stiffness mode, the robotic hand stiffness is electronically mapped from the operator's grip stiffness.

The operator's grip stiffness is acquired using surface electromyography (sEMG) of intrinsic hand muscles according to Höppner et al. (2017), a method which is known in literature as “teleimpedance.” One of the most prominent studies focusing on teleimpedance was conducted by Ajoudani et al. (2012) who teleoperated the Cartesian stiffness of a lightweight robot during a peg-in-hole and a ball-catching task using sEMG of eight arm muscles and visual feedback. Both tasks were performed better with the teleimpedance control (i.e., reduced positional errors and less force exertion) compared to a constantly low- or high-stiffness control, which demonstrates the effectiveness of this method for impedance-controlled robotic arms.

Teleimpedance using sEMG has also been studied for VSA hands (Hocaoglu and Patoglu, 2012; Godfrey et al., 2013; Ajoudani et al., 2014). In these studies, the sEMG signal was acquired from the extrinsic hand muscles in the forearm. Godfrey et al. (2013) found that adding impedance control and vibrotactile feedback to a teleoperation setting improved the user experience and reduced the physical and mental effort when grasping objects. Laghi et al. (2017) investigated the role of force feedback, visual feedback and communication delay in a teleimpedance approach, as well. The aim of their study was to verify the usefulness of a combined teleimpedance mode with force feedback in comparison to standard control modes. They found their newly introduced method to be working even when in the presence of a communication delay.

The present study builds upon the existing teleimpedance studies, but differs in some aspects. Firstly, the sEMG signals of intrinsic instead of extrinsic hand muscles are measured, as they have been shown to provide a more accurate estimate of grip stiffness (Höppner et al., 2017). The better signal may be due to lower electrical resistance and less cross-talk from other muscles, which both lead to a better signal-to-noise ratio.

Secondly, grip forces are fed back to the operator as grip forces instead of vibrotactile stimuli. We assume that this more intuitive feedback enables the operators to better adjust the stiffness more effectively. Thirdly, the hand is positioned in space by the human arm via an attached beam instead of via a robotic arm. This is mainly done to simplify the experiment setup.

Like the robotic VSA hand, the human hand can be considered a variable-stiffness mechanism (Höppner et al., 2011, 2017). Generally speaking, a higher contractile muscle force will result in a higher stiffness. Through co-contracting antagonistic muscle parts the human is able to decouple force from stiffness and to increase stiffness without changing joint torque (McIntyre et al., 1996; Perreault et al., 2002, 2004) by about 20% for the human hand (Höppner et al., 2017).

Because of this familiarity with variable stiffness and since humans are known to adapt very well to new situations, we assume that in the present task—after trials of learning—the human operators will outperform any existing robotic controller. Hence, the research questions are focused not primarily on the controller, but on the robotic hand hardware, the stiffness mode and the level of sensory feedback.

Section 2 describes the experiment materials and methods. It introduces the design of the WHISG hand—a new soft robotic hand based on VSA technology—, briefly describes the acquisition of sEMG signals for teleoperating the robotic hand stiffness and explains the experimental and statistical design in detail. In section 3, the results are listed. The influence of the factors force and visual feedback as well as stiffness mode on the three metrics—duration to complete one trial, number of single grasping actions and required thumb torque—is presented. Finally, section 4 discusses the results in the context of the research question, i.e., the effect of stiffness modes and sensory feedback modes on grasp performance and on the stiffness modulation strategy and draw possible conclusions and implications for soft robotics.

## 2. Materials and methods

The comparison of SEA and VSA grasping under different levels of sensory feedback entailed the design of a suitable robotic hand, interfacing it to the human operators, setting up the experimental environment and measuring the grasping performance during different experimental conditions.

### 2.1. Design of the WHISG hand

**Figure 1:**
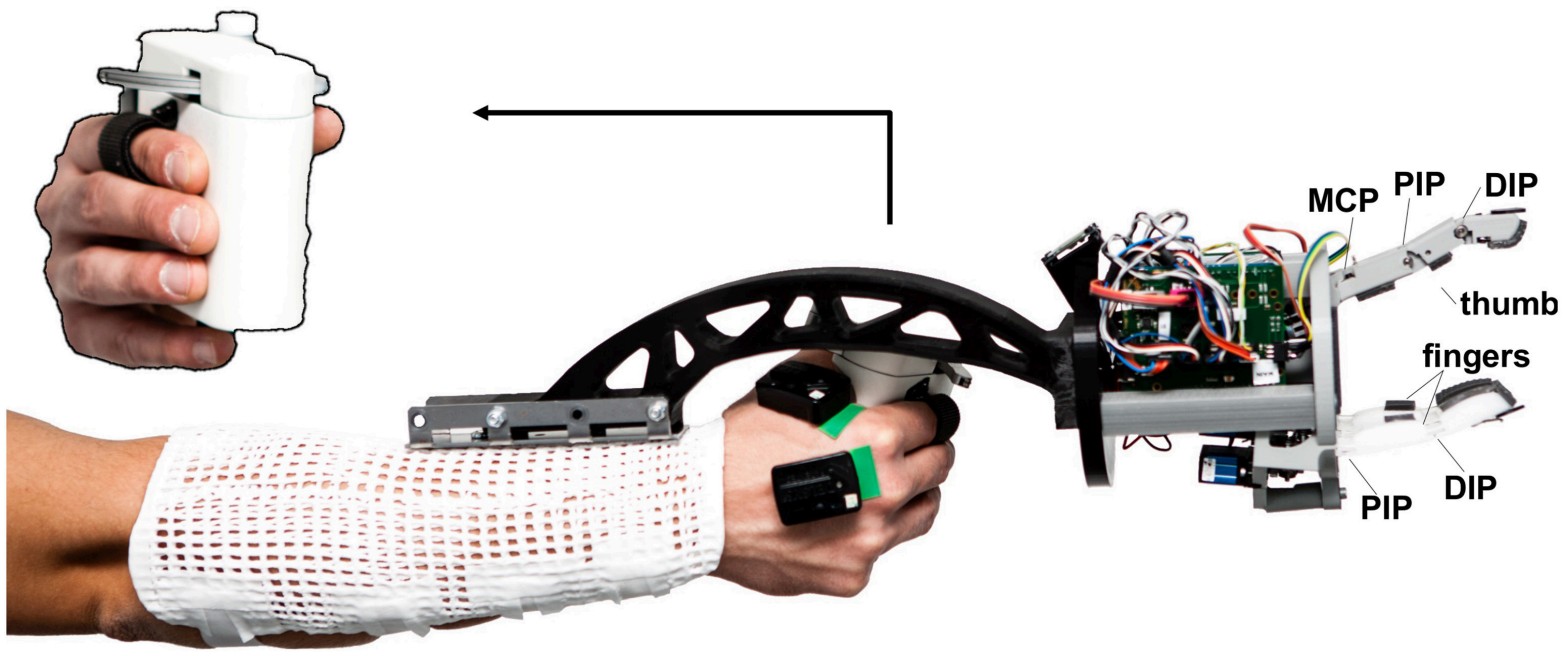
DLR WHISG hand mounted on the forearm of the operator with a supporting splint and two sEMG electrodes placed on two intrinsic hand muscles. Top left: a better view on the Grip Force Master.

**Figure 2:**
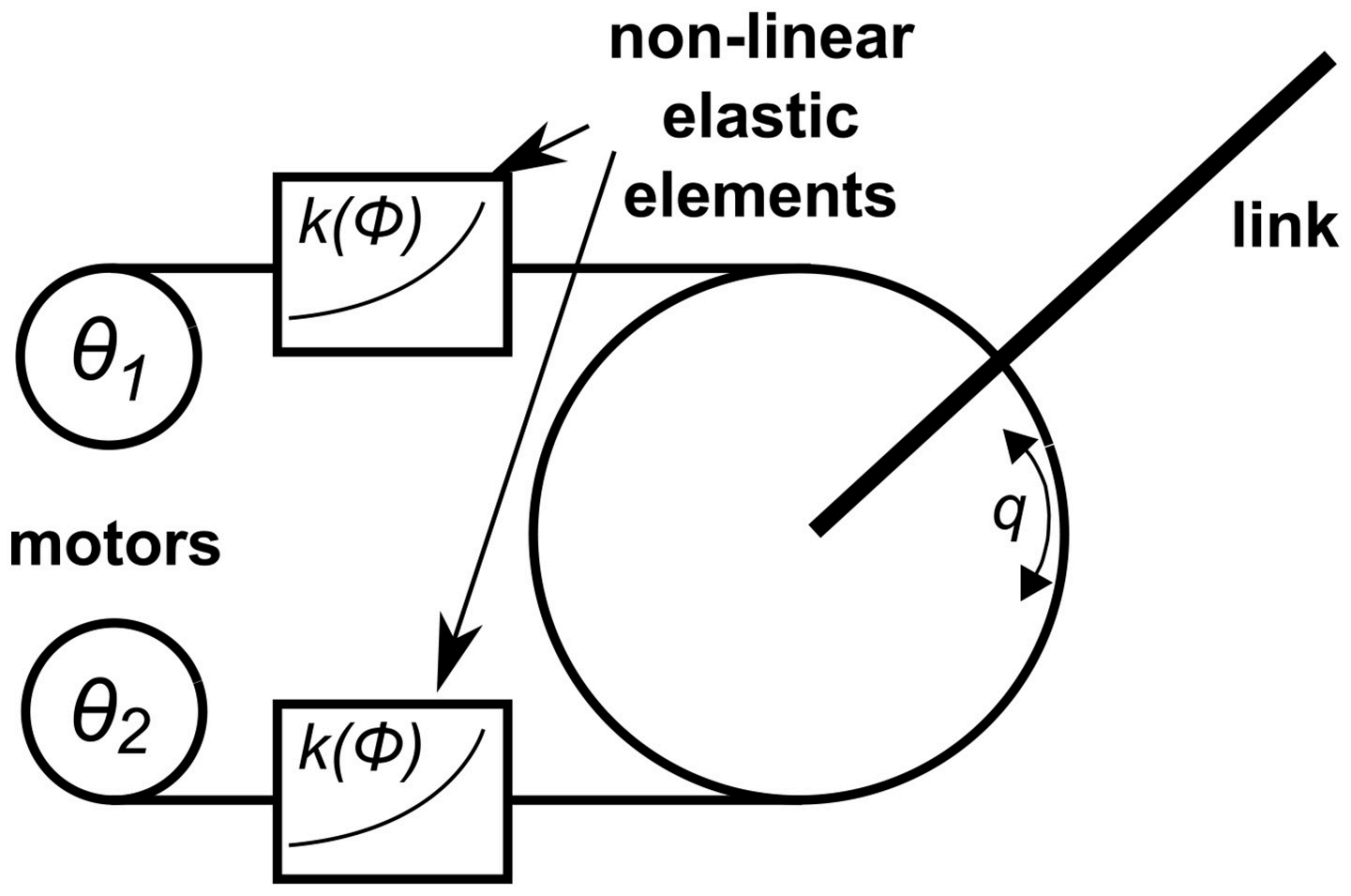
Schematic illustration of the FAS mechanism. Antagonistic actuation mechanism with two motors, non-linear elastic elements, and a joint link. The angle θ_*i*_ denotes the motor position, *k*(ϕ) is the spring stiffness depending on the spring deflection ϕ, *q* is the joint angle. With kind permission of Grebenstein (2012).

The WHISG hand, at only 500 g, is light enough to be hand-held or mounted on a lightweight robot or, as is the case in the present study, on the forearm of an operator (see Figure 1 and Video 1). The hand is designed to study grasping a broad variety of delicate groceries, such as cucumbers, mangoes, small-fruit containers, salad etc. However, for this first version the kinematic design is roughly estimated rather than based on a detailed kinematic analysis. It is a three-fingered hand with inherent compliance due to elastic elements between motor and joint (see Figure 2), enabling shape adaptation and safe interaction with the environment. We choose three instead of two fingers in order to increase the grasp stability as well as to ensure a more evenly distribution of applied object forces. The housing parts are 3D-printed from polylactic acid filament. Motor control is done with two Arduino Leonardo micro-controllers that communicate using their serial peripheral interface.

The hand contains two types of fingers: one thumb-like main finger with four degrees of freedom (DoF) and two underactuated fingers with two DoF each, which oppose the thumb. The two thumb DoF closest to the base constitute the metacarpophalangeal (MCP) joint. It allows sideways (adduction/abduction) and bending (flexion/extension) movement. The MCP is followed by the proximal interphalangeal (PIP) and distal interphalangeal (DIP) joints with one flexion/extension DoF each. The opposing fingers contain only one PIP and one DIP joint each.

For adjusting the stiffness of the joints, the Flexible Antagonistic Spring (FAS; Friedl et al., 2011) mechanism of the DLR Hand Arm System (Grebenstein et al., 2011) is used (Figure 2). The joint is connected to two tendons, each of which can move it in one of two opposing directions (e.g., flexion and extension). The tendons each run to electric servo motors via elastic elements with non-linear force-displacement characteristics, where the force is a convex, increasing function of the displacement. The convexly increasing force-displacement characteristics cause the stiffness of the elastic element to rise when the tendon force increases. Due to the antagonistic arrangement of the tendons, the stiffnesses of both tendons add up while the forces cancel each other. Thereby, the stiffness of the joint can be varied independently from the joint torque. The difference in the actuation of the motors defines whether position or stiffness of the joint is changed. Motion at the joint can be achieved by moving both servos in the same direction, changing the stiffness by moving in opposite directions. The maximum joint torque is limited by the maximum motor torque because each motor can only apply forces in one direction (Grebenstein, 2012).

**Figure 3:**
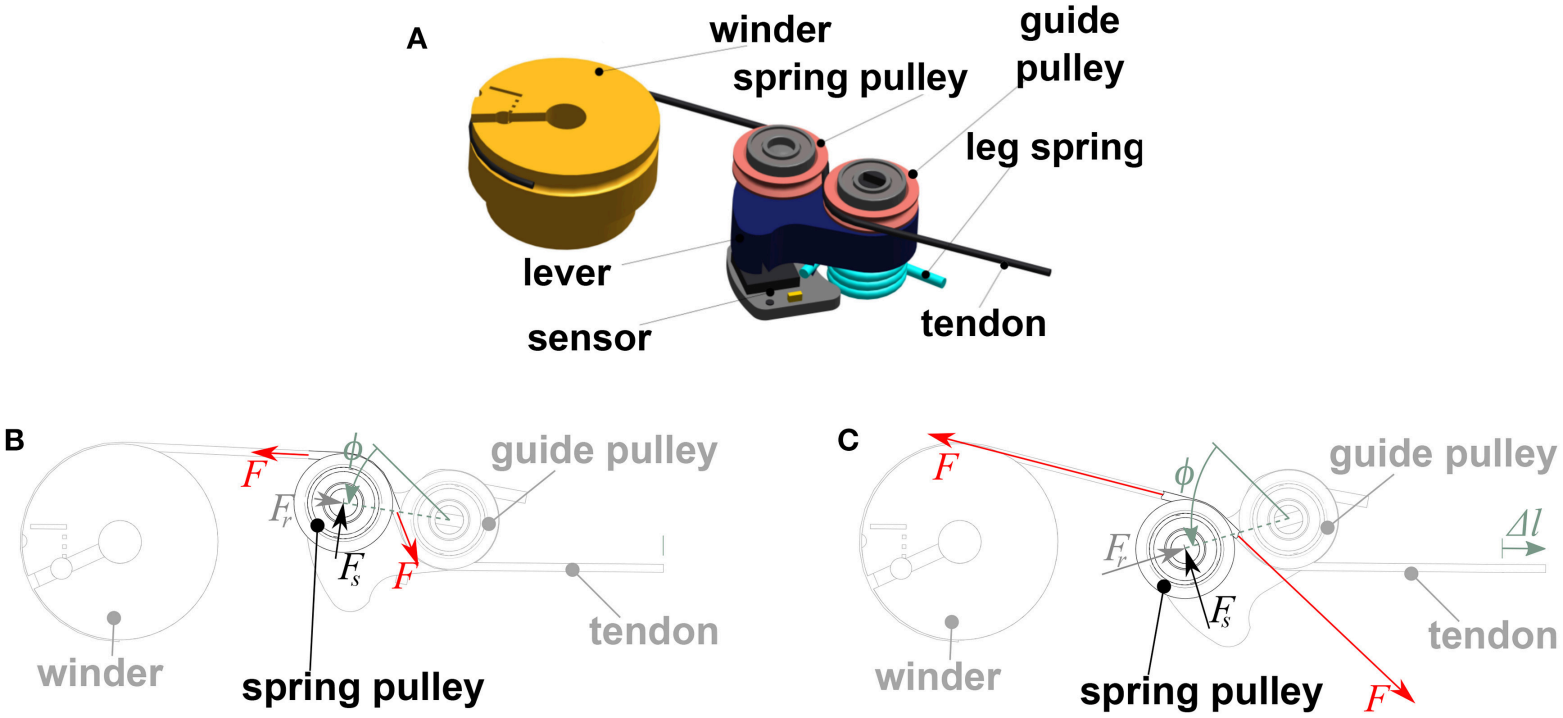
**(A)** Non-linear elastic element of the WHISG FAS consisting of a guide pulley, a leg spring, a lever, and a spring pulley. **(B)** Soft configuration. **(C)** Stiff configuration. An increase of Δ*l* of the tendon excursion leads to an increase of the spring angle ϕ, which leads to a higher spring force *F*_*s*_ acting on the spring pulley via the lever. The relationship between spring force *F*_*s*_, bearing reaction force *F*_*r*_ and tendon force *F* results from an equilibrium around the spring pulley. As the spring angle ϕ increases, the portion of the tendon forces *F* that points radially to the center of the spring pulley decreases and therefore the forces increase progressively to keep counteracting the spring and bearing reaction forces.

Figure 3 shows the mechanical design of the elastic elements and how the change of the angles between the tendons and the lever lead to the convexly increasing force-displacement characteristics. Initial stiffness depends on the position of the spring and its constant as well as on the distance between winder and spring pulley (Friedl et al., 2011). Tensioning the tendon by actuating the winder increases the spring deflection angle ϕ. Said angle is measured by hall sensors (iC-MP sensors by iC-Haus) and custom-made magnets, and is used to calculate *F*_*s*_, the force exerted on the spring pulley.

Having two motors per DoF (2*N*, where *N* is the number of DoF) allows setting the stiffness of each joint separately from the other joints. This concept is followed, e.g., in the DLR Hand Arm System. To reduce weight and cost, the WHISG hand uses less than 2*N* motors.

**Figure 4:**
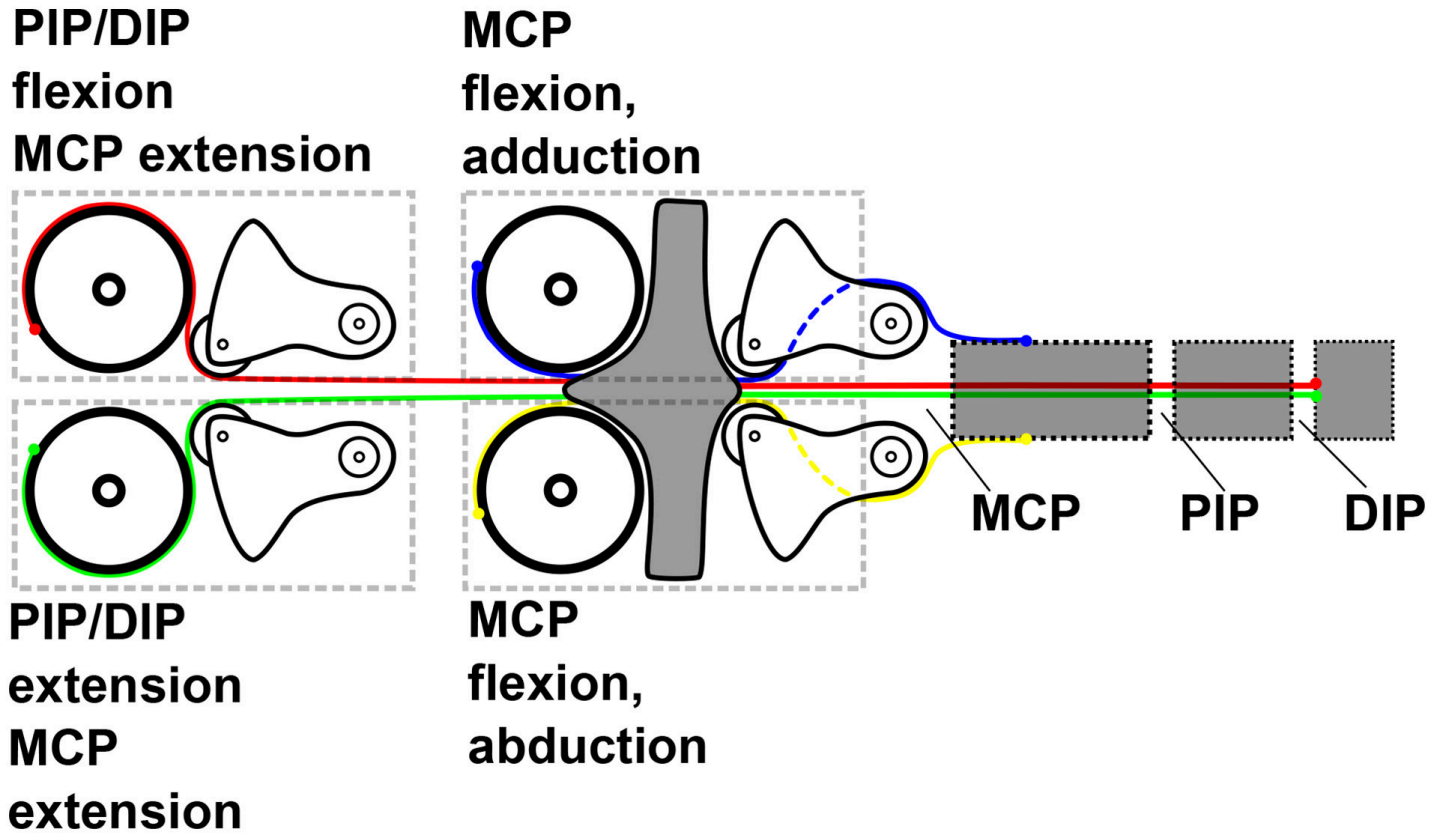
Tendon routing and motor functions of the WHISG thumb (viewed from the palmar side).

The four-DoF thumb is actuated by four Bluebird BMS 385 servo motors (Figure 4). The PIP joint and the DIP joint share one set of tendons and move differentially, i.e., only the sum of the movement can be controlled. The DIP joint is equipped with an extension spring so that in the absence of external forces only the PIP joint moves. The DIP joint only comes into play when the PIP joint comes into contact with an object and cannot move any further, therefore ensuring a hook-like grip on the object. A change in stiffness affects all four thumb joints at the same time, i.e., the whole thumb can be made stiffer or softer, but not single joints. The number of motors is thus *N* = *N*−1+1, where −1 stands for the underactuation of the PIP and DIP joints and +1 stands for the ability to vary one common stiffness value. The four motors each provide a maximum torque of 0.45 Nm resulting in a maximum force at the fingertip of around 10 N.

**Figure 5:**
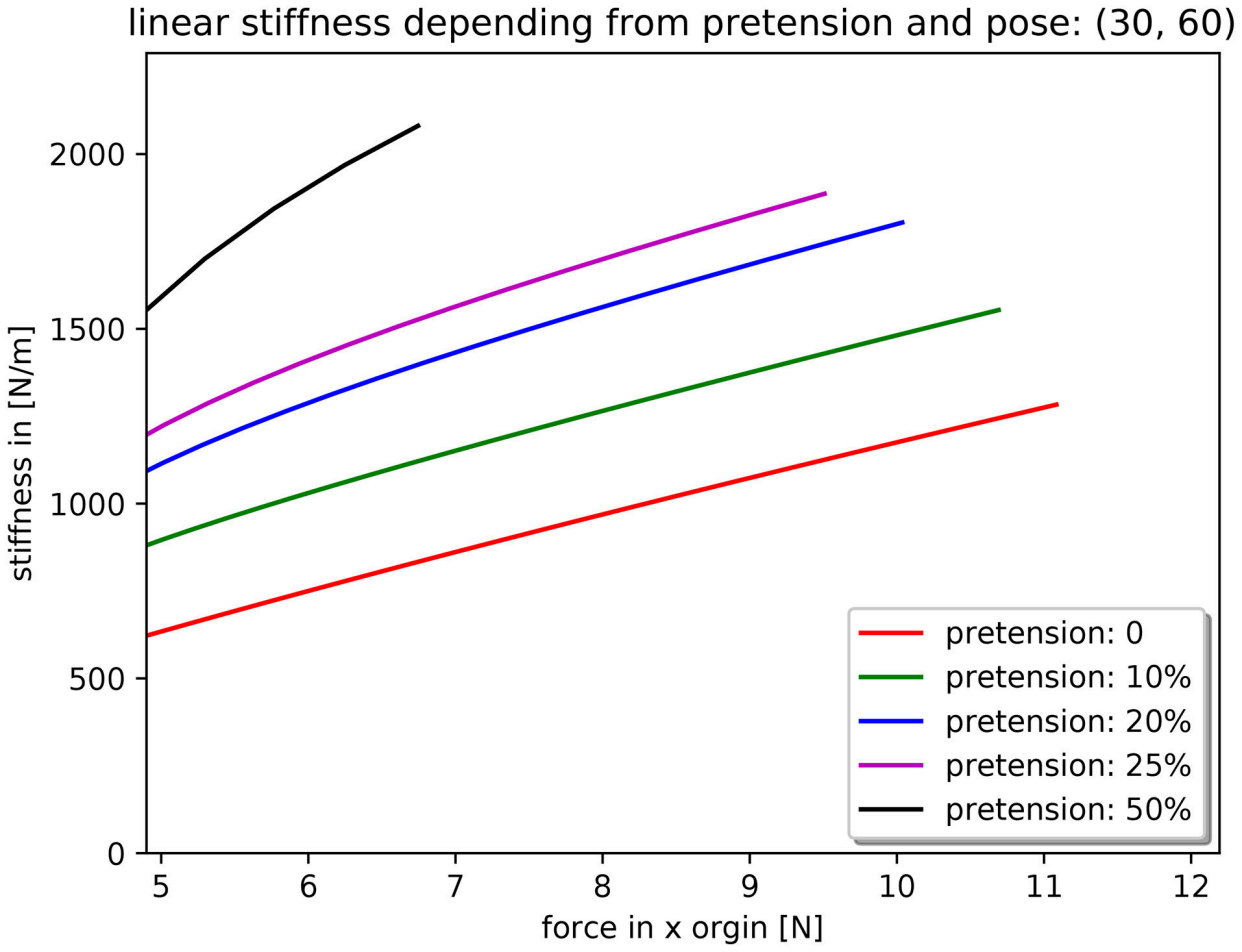
Stiffness behavior of the thumb tip as a result of the flexible antagonistic spring mechanisms in the thumb joints and their pretension.

The resulting stiffness behavior at the thumb tip is exemplarily shown in Figure 5 for a linear excursion of the thumb tip from a posture of 30° flexion of the MCP joint and 60° flexion of the PIP and DIP joints. Increasing the pretension of the antagonistic tendons of the joints leads to a shift in the force-stiffness relationship.

**Figure 6:**
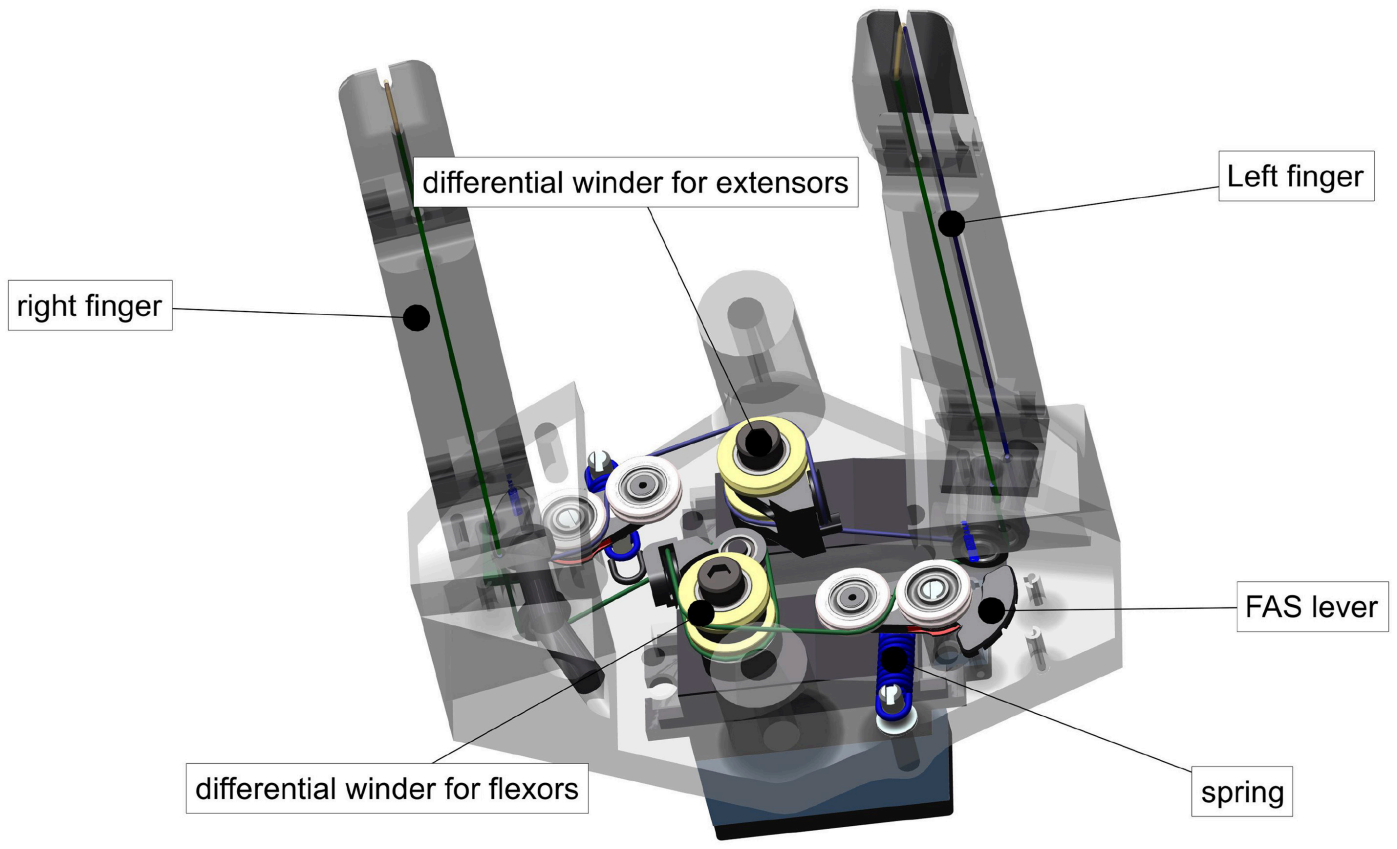
Mechanical design of the WHISG fingers including actuation.

The two fingers with two DoF each are even more underactuated by only two (*N*/2−1+1) Bluebird BMS 390 DMH servo motors together (Figure 6). Each servo motor actuates one of the differential winders, each of which in turn differentially actuates one movement direction of both fingers, thereby dividing the required number of motors by two. Furthermore, one motor is saved by the combined actuation of PIP and DIP. For tuning the common stiffness of the two fingers, an additional motor is used.

### 2.2. Interfacing the robotic hand to the human operator

For positioning and moving around the WHISG hand, it was physically attached to the right forearm of the human operator via a beam and a splint (Figure 1).

The gripper opening angle was controlled by the operator's thumb and index finger via the Grip Force Master (GFM) by Force Dimensions (Figure 1 top left). It basically consists of a small lever arm tendon-coupled to an electrical motor, which sets the distance between human index finger and thumb. Force-control is achieved by measuring the motor current. In the force feedback mode, the GFM could feed back the grip force of the WHISG hand to the human hand.

In the variable-stiffness mode, the pretension of the WHISG thumb and fingers, and thereby their stiffness behavior, was controlled via the operator's grip stiffness, which was acquired via sEMG signals from intrinsic hand muscles according to Höppner et al. (2017). To measure the sEMG signals, wireless Trigno Standard Sensor electrodes from Delsys Inc. were used in connection with a Trigno Lab base station. These electrodes comply with the requirements put forth by the Medical Device Directive 181 93 / 42 / EEC, and the experiment complied with their intended use. The sEMG signals were processed on a custom-designed low-cost analysis box based on an Arduino Duo microcontroller board. The signals were band-pass filtered by a Butterworth 2nd order filter with a cutoff frequency of 20–500 Hz, rectified by root mean square and smoothed using a moving-average filter with a window size of 75 ms. Furthermore, the signals were calibrated to the individual operator by subtracting the baseline noise (recorded while resting the muscles) and divided by a signal recorded during maximum voluntary muscle contraction. The sum of the calibrated sEMG signals was mapped to the range of available pretension levels of the WHISG joints, which modify its stiffness behavior.

**Figure 7:**
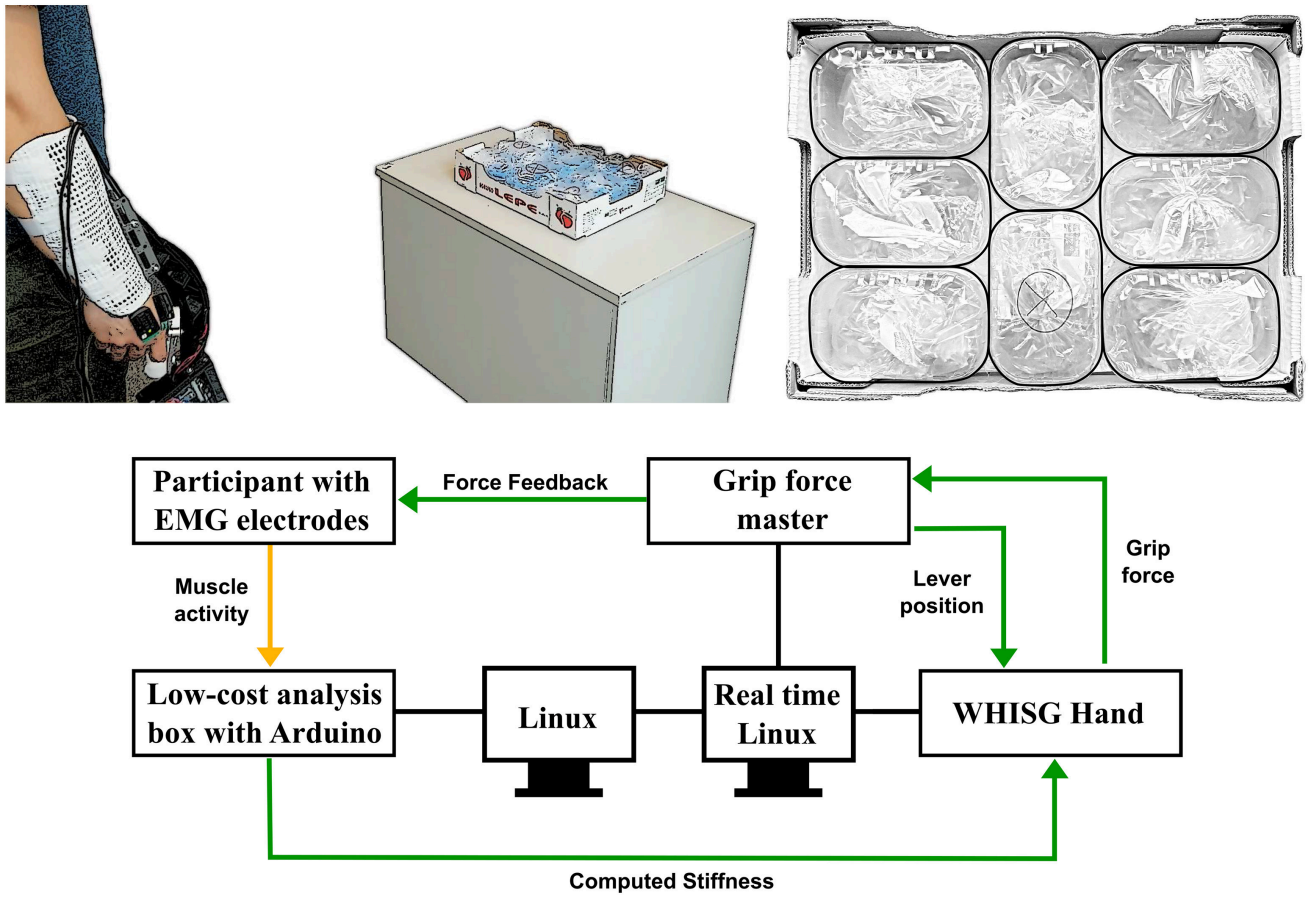
**(Upper left)** Experimental setup with the fastened WHISG hand and the placed electrodes on the back of the operator's hand as well as the cardboard box placed on the table. **(Upper right)** Single plastic boxes arranged in the cardboard box; middle cardboard box marked with “x” had to be grasped (black border retroactively inserted). **(Bottom)** Block diagram representing signal flow within the experimental setup.

A block diagram depicting the connections between the subsystems is shown in Figure 7 (bottom). The sEMG electrodes send continuously analog data (yellow arrow) to the low-cost analysis box. The calculated stiffness is send to the Linux PC using the serial port of the Arduino microcontroller. The Linux, the GFM, and the WHISG hand are connected to a Linux real-time PC running Matlab Simulink which ensures a proper synchronization between sEMG electrodes, force feedback device and robotic hand (green arrows demonstrate real-time signals). The grip force is measured using the deflection of the springs of the WHISG hand and fed back to the GFM. Subjects noticed higher grip forces through an increased resistance for pressing the GFM lever arm. The lever arm position was sent back to the WHISG hand for controlling its grasp width. An increase in stiffness results in an overall pretension of all 6 FAS elements of thumb and fingers (see section 2.1).

### 2.3. Experimental setup

Six healthy subjects, all male, five right-handed and one left-handed, age 24–30, all naive to the experiment, took part as operators and performed the experimental protocol described below. The whole procedure lasted between 60 and 75 min per participant. Oral and written descriptions of the experiment were provided to the subjects. After all questions were answered a written consent form was signed by all participants. These experiments are compliant with the World Medical Association's Declaration of Helsinki, regarding the ethical principles for medical research involving human subjects, last version, as approved at the 59th WMA General Assembly, Seoul, October 2008. Necessary approvals for the subject studies were received from the organization-wide works council of the German Aerospace Center as well as its institutional board for data privacy. A physician is part of the works council. The collection and processing of experimental data were approved by both committees.

#### 2.3.1. Task: object grasping with different stiffness settings

Operators were asked to grip one out of eight tightly packed small-fruit containers (LxWxH: 140 mm x 90 mm x 75 mm) out of a cardboard box (LxWxH: 400 mm x 300 mm x 90 mm) using the WHISG hand (see Figure 7 and Video 1). The fruits were replaced with water-filled plastic bags to simulate their weight, 125 g. The cardboard box was placed on a waist-high table and fixed with adhesive tape such that no movement of the box was possible. One single grasping action was conducted as follows: The operator started approximately two steps away from the table to hinder learning of the optimal, initial grasping position. The WHISG hand was held on the right side of the operator's body, pointing downwards. With the start signal of the experimenter, the operator walked up to the table upon which data acquisition started and the operator began acquiring a grasp of the container. After successfully grasping the container, operators were asked to lift it up and place it right next to the box. After placing it, operators had to take two steps back and return to the starting position, which was the trigger to end data acquisition. Afterwards the plastic box was placed back into the cardboard box by the experimenter before the next grasping action will be performed.

#### 2.3.2. Trials with different combinations of force feedback, visual feedback and stiffness mode

Each participant conducted 60 grasping trial in total: 12 were training trials to minimize learning effects for the remaining 48, which were used for statistical analysis. Every 12 trials, operators were asked if they wanted to take a small break to minimize fatigue. In each of the 12-trials sub blocks, all combinations of stiffness modes and feedback modes were tested.

The stiffness modes consisted of variable stiffness (VS), low fixed stiffness (LS), and high fixed stiffness (HS). VS corresponded to the range of 0 to 55% pretension of the FAS mechanisms, LS to 5% pretension and HS to 55% pretension. Each subject was assigned a different permutation of stiffness modes to reduce effects of learning or fatigue on the results.

Within each stiffness mode, the following feedback modes were tested:

- visual feedback (VF) only: the operator had an unimpaired view of the experiment area and the force generation of the GFM was switched off;
- no feedback (NF): the visual feedback was impaired by altered welding goggles and the force generation of the GFM was switched off; the welding visor glasses were replaced with plastic disks which were processed with sandpaper and an added adhering plastic sheet; objects and their outlines seen through these glasses were blurred;
- full feedback (VF+FF): the operator had an unimpaired view of the experiment area and the force generation of the GFM was switched on;
- force feedback (FF) only: the visual feedback was impaired and the force generation of the GFM was switched on.

A questionnaire (5-point Likert scale) was filled about the usefulness of the different stiffness modes, about exploiting the environment to get a secure grasp on the object and about the helpfulness of obtaining force feedback.

### 2.4. Statistical design

To answer the research questions and evaluate the hypotheses of the study, several types of data were collected and analyzed during the trials.

For investigating the effects of stiffness and feedback modes on the grasp performance and for evaluating the hypothesis that variable stiffness helps to compensate lack of sensory feedback better than fixed stiffness, four performance measures were recorded:

- the grasp success rate; a grasp was counted as successful whenever the small-fruit container was grasped, lifted, and placed on the table next to the cardboard box within the time limit of 2 min; the grasp success rate was calculated for each experimental condition (i.e., for each combination of stiffness and feedback modes) as the number of trials with grasp success divided by the total number of trials within this experimental condition over all operators and repetitions;
- the task completion time of each trial, i.e., in case of successful grasps, the time it took to complete the task, and otherwise, the 2 min after which the trial was aborted;
- the number of grasping attempts for each trial, i.e., the number of times that the commanded gripper opening angle crosses a certain threshold and thereby starts a gripping phase;
- the mean thumb gripping torque for each trial, i.e., the mean torque of the thumb joints during the last gripping phase before placing the container on the table.

For the grasp success rate, 12 values for twelve the experimental conditions were recorded, while the other three recorded performance measures contained 288 values for 288 trials.

The three latter performance criteria were analyzed with a linear mixed regression model,

(1) $$\begin{array}{l} y_{ijkmn}=\beta_{0}+\beta_{sti\mathrm{ff}ness\text{\_}mode,i}+\beta_{visual\text{\_}feedback,j}\\ +\beta_{force\text{\_}feedback,k}+\beta_{sti\mathrm{ff}ness\text{\_}mode\times visual\text{\_}feedback,ij}\\ +\beta_{sti\mathrm{ff}ness\text{\_}mode\times force\text{\_}feedback,ik}\\ +\beta_{visual\text{\_}feedback\times force\text{\_}feedback,jk}\\ +\beta_{sti\mathrm{ff}ness\text{\_}mode\times visual\text{\_}feedback\times force\text{\_}feedback,ijk}\\ +m\beta_{trial\text{\_}number}\epsilon_{operator,n}+\epsilon_{mn}, \end{array}$$

where *y*_*ijkmn*_ is the response variable, i.e., any of the three above-mentioned performance measures—task completion time, number of grasping attempts or mean thumb gripping torque—, *i* is the stiffness mode, *j* denotes the presence of unimpaired visual feedback, *k* denotes the availability of gripping force feedback, *m* is the within-operator trial number, *n* is the operator number, β_0_ is the intercept, which is a constant term, β_stiffness_mode,*i*_ is the fixed effect of the stiffness mode, β_visual_feedback,*j*_ is the fixed effect of visual feedback, β_force_feedback,*k*_ is the fixed effect of force feedback, β_stiffness_mode×visual_feedback,*ij*_, β_stiffness_mode×force_feedback,*ik*_, β_visual_feedback×force_feedback,*jk*_, and β_stiffness_mode×visual_feedback×force_feedback,*ijk*_ are the fixed effects of their interactions, *mβ*_trial_number_ is the trial-number-dependent fixed effect of learning or fatigue, ϵ_operator,*n*_ is the operator-specific random effect and ϵ_*mn*_ is the residual random error. The random effects are assumed to follow normal distributions as follows:

(2) $$\begin{array}{l} \epsilon_{mn}\overset{i.i.d.}{\sim }N\left(0,\sigma^{2}\right)\text{and} \end{array}$$

(3) $$\begin{array}{l} \epsilon_{operator,m}\overset{i.i.d.}{\sim }N\left(0,\tau^{2}\right). \end{array}$$

How operators experienced the helpfulness of the different stiffness modes and the availability of force feedback was measured using a questionnaire (5-point Likert scale) with three questions for the stiffness modes and one question for force feedback. The answers were grouped into positive, neutral, and negative groups and reported summarily over all operators.

For the analysis of the stiffness modulation strategies in the variable-stiffness mode, the mean normalized stiffness values in the searching phase and the grasping phase were recorded.

They were analyzed using the following mixed regression model:

(4) $$\begin{array}{l} K_{mean,jklmn}=\beta_{0}+\beta_{visual\text{\_}feedback,j}+\beta_{force\text{\_}feedback,k}+\beta_{phase,l}\\ +\beta_{visual\text{\_}feedback\times phase,jl}+\beta_{force\text{\_}feedback\times phase,kl}\\ +\epsilon_{operator,n}+\epsilon_{mn}, \end{array}$$

where *K*_mean,*jklmn*_ is the response variable, i.e., the mean normalized stiffness, *l* denotes the phase (searching or gripping phase), β_phase,*l*_ its fixed effect on the response variable, β_visual_feedback×phase,*jl*_ and β_force_feedback×phase,*kl*_ its interactions with the feedback modalities and the other variables as in Equation (1).

The parameters of the mixed models were fitted to the measured outcome measures using the *lmer* function of the *lme4* library (Bates et al., 2015) of the R statistics software (R Core Team, 2015).

The exploitation of environmental constraints was observed by the experimenter, classified and reported summarily. The response of operators to the questionnaire whether they used the environment to obtain a secure grasp on the object was reported summarily.

## 3. Results

The main results of the experiments consist of (a) the effects of stiffness and feedback modes on the grasp performance and user experience, (b) in the case of the variable-stiffness mode, observations of stiffness modulation strategies, and (c) observations of strategies for exploiting environmental constraints.

The effect of the trial number, which can account for learning or fatigue, is two to three orders of magnitude smaller than the effects of stiffness and feedback.

### 3.1. Effects of stiffness and feedback modes on the grasp performance

The grasp performance is measured by four criteria: grasp success rate, task completion time, number of grasp attempts, and mean gripping torque.

The grasp success rate is 100% for all stiffness and feedback modes.

**Figure 8:**
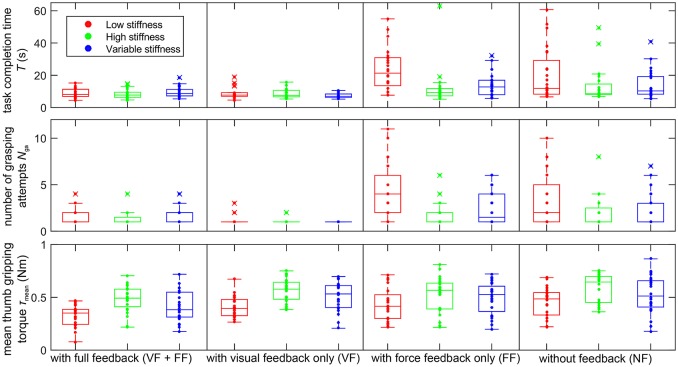
Measured values (samples and box plots) of task completion time *T* **(Top)**, number of grasping attempts *N*_ga_ **(Middle)** and mean thumb gripping torque τ_mean_ **(Bottom)** of all operators and trials with low (red), high (green), and variable stiffness (blue). One extreme sample of τ_mean_ of 3.7 Nm lies outside the plotting range.

For the other three grasp performance measures—task completion time *T*, number of grasp attempts *N*_ga_, and mean gripping torque τ_mean_—, the absolute measurement values at the different combinations of stiffness and feedback modes are shown as samples and box plots in Figure 8. The task completion time ranges from 4 to 63 s, the number of grasping attempts from 1 to 11 and the mean thumb gripping torque from 0.07 to 0.86 Nm, plus one outlier at 3.67 Nm, which could not be explained and remained in the data set for the analysis. For all three measures, low values are desirable: lower task completion times enable higher productivity, a lower number of task attempts indicates higher reliability and lower gripping torques indicate gentler handling of the manipulated goods.

**Figure 9:**
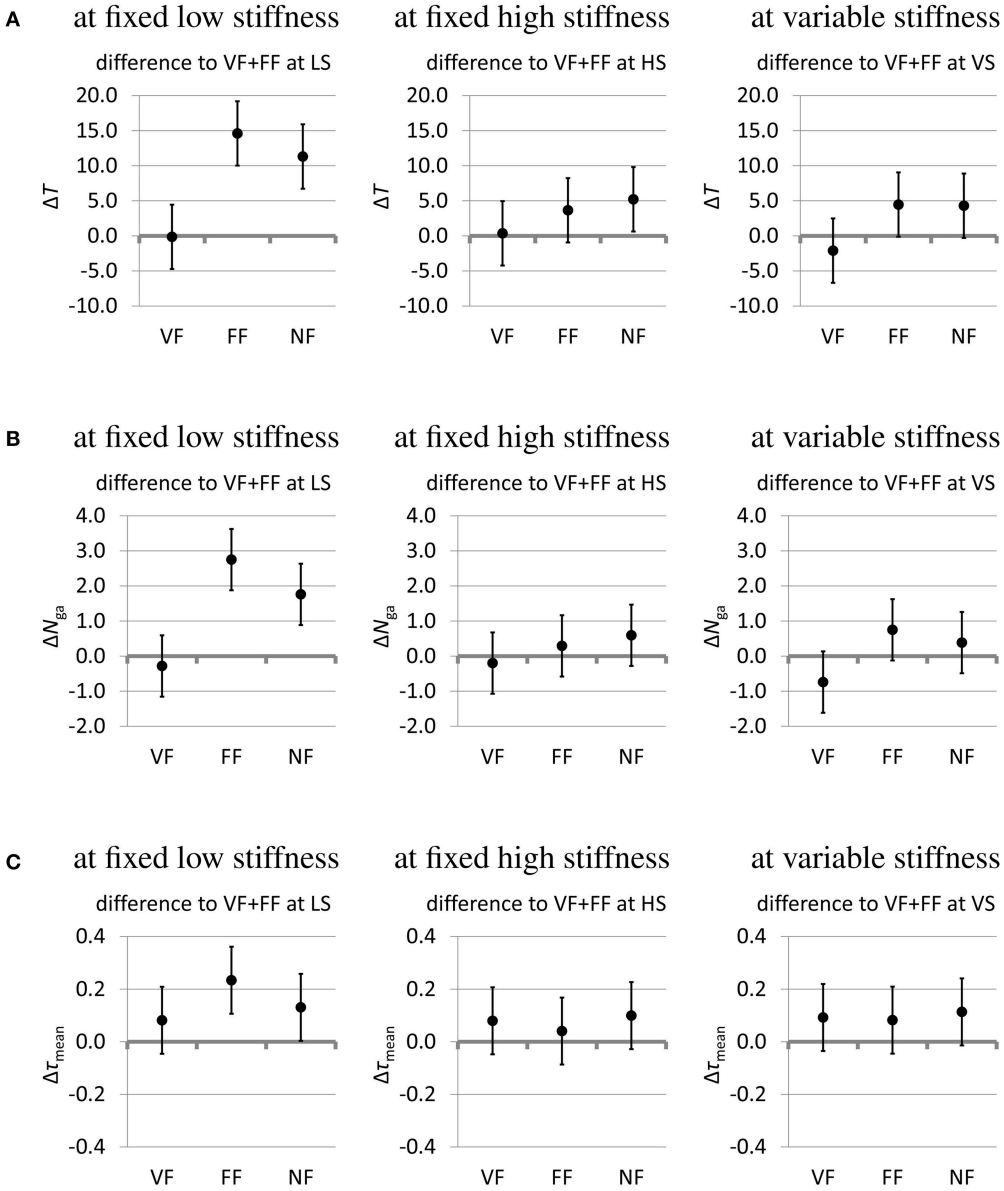
**(A)** Effect of sensory feedback modes on the time *T*(s) to complete the task. **(B)** Effect of sensory feedback modes on the number *N*_ga_ of grasp attempts. **(C)** Effect of sensory feedback modes on the mean thumb torque τ_mean_(Nm). The effect of the sensory feedback modes on the outcome measures (estimates and 95% confidence intervals from fitting the mixed model). Visual feedback only (VF), force feedback only (FF), and no feedback (NF) are compared to the baseline of full feedback (visual+force feedback, VF+FF). For all outcome measures, low values are desirable.

**Figure 10:**
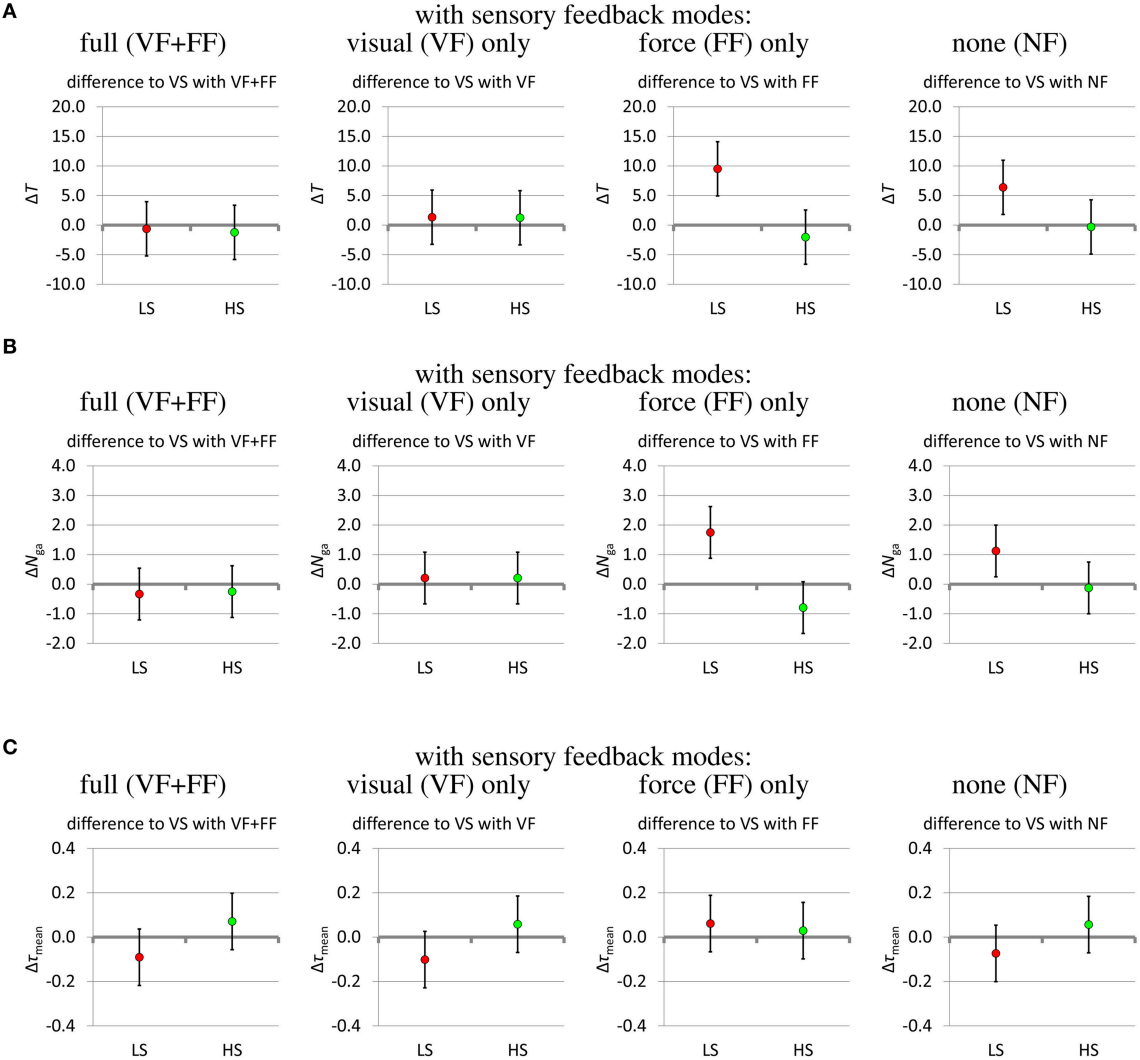
**(A)** Effect of stiffness modes on the time *T*(s) to complete the task.**(B)** Effect of stiffness modes on the number *N*_ga_ of grasp attempts.**(C)** Effect of stiffness modes on the mean thumb torque τ_mean_(Nm). The effect of the stiffness modes on the outcome measures (estimates and 95% confidence intervals from fitting the mixed model). Fixed low stiffness (LS) and fixed high stiffness (HS) are compared to the baseline of variable stiffness (VS). For all outcome measures, low values are desirable.

How these three measures are affected by the presence or lack of sensory feedback and by the variable or fixed grip stiffness modes is shown in Figures 9, 10 as 95% confidence intervals of the results of the fitted mixed model of Equation (1). Whenever the confidence interval does not include zero, the effect is statistically significant at a significance level of α = 0.05. In some cases of a slight overlap between the confidence interval and the zero level we carefully speak of *tendencies*. Since the significance level is not corrected for multiple comparisons, the confidence intervals are interpreted in conjunction with each other as aggregate results, rather than independently as separate results.

The distributions of the residuals of the mixed models of task completion time and number of grasping attempts were somewhat positively skewed compared to a normal distribution, but still unimodal and smooth. While the method is robust to deviations from the normal distribution, this adds some uncertainty to the results.

The effect of the sensory feedback is shown in Figure 9. Different sensory impairments are compared against the baseline of full sensory feedback (VF+FF). Taking away the grip force feedback leaves visual feedback (VF) remaining. Conversely, impairing the visual feedback leaves grip force feedback (FF) remaining. Taking away both the force feedback and impairing the visual feedback leads to the “no-feedback” (NF) mode, where the remaining feedback is actually limited to blurred visual feedback as well as auditory feedback and some direct force feedback via the splint on the forearm, which are present in all feedback conditions. Lack of visual feedback deteriorates all performance measures in the low-stiffness mode (Δ*T* ≈ +11 s…+15 s, Δ*N*_ga_ ≈ +2…+3, Δτ_mean_ ≈ +0.1 Nm… +0.2 Nm; Figures 9A–C left column, FF and NF) and shows a tendency of slightly deteriorating them in the other two stiffness modes (Δ*T* ≈ +4 s…+5 s, Δ*N*_ga_ ≈ +0.2…+0.8, Δτ_mean_ ≈ +0.04 Nm… +0.1 Nm; Figures 9A–C middle and right columns, FF and NF). Lack of force feedback shows a tendency to deteriorate the gripping torque (Δτ_mean_ ≈ +0.08 Nm… +0.15 Nm; Figure 9C VF and NF). In the absence of visual feedback and when using the low-stiffness mode, lack of force feedback shows a slight tendency to improve the performance criteria (Δ*T* ≈ −3 s, Δ*N*_ga_ ≈ −1, Δτ_mean_ ≈ −0.05 Nm; Figures 9A–C left column, NF vs. FF).

Figure 10 shows the effect of the stiffness modes on the grasping performance. The fixed-stiffness modes low stiffness (LS) and high stiffness (HS) are compared against the baseline of variable stiffness. In the absence of visual feedback, low fixed stiffness increases the task completion time on average by about 6 to 10 s (Figure 10A) and the number of grasp attempts by about 1.1 to 1.8 (Figure 10B). High fixed stiffness shows a slight tendency to increase the gripping torque by about 0.02 to 0.08 Nm (Figure 10C HS), while low fixed stiffness shows a tendency to reduce the gripping torque by about 0.06 to 0.08 Nm, except in the presence of force feedback only (Figure 10C LS).

In the questionnaire, operators gave one neutral and five negative responses for the low stiffness, five positive and one neutral for the high stiffness and four positive and two neutral for the variable stiffness setting. Four out of six operators found the presence force feedback helpful.

### 3.2. Observations of stiffness modulation strategies

**Figure 11:**
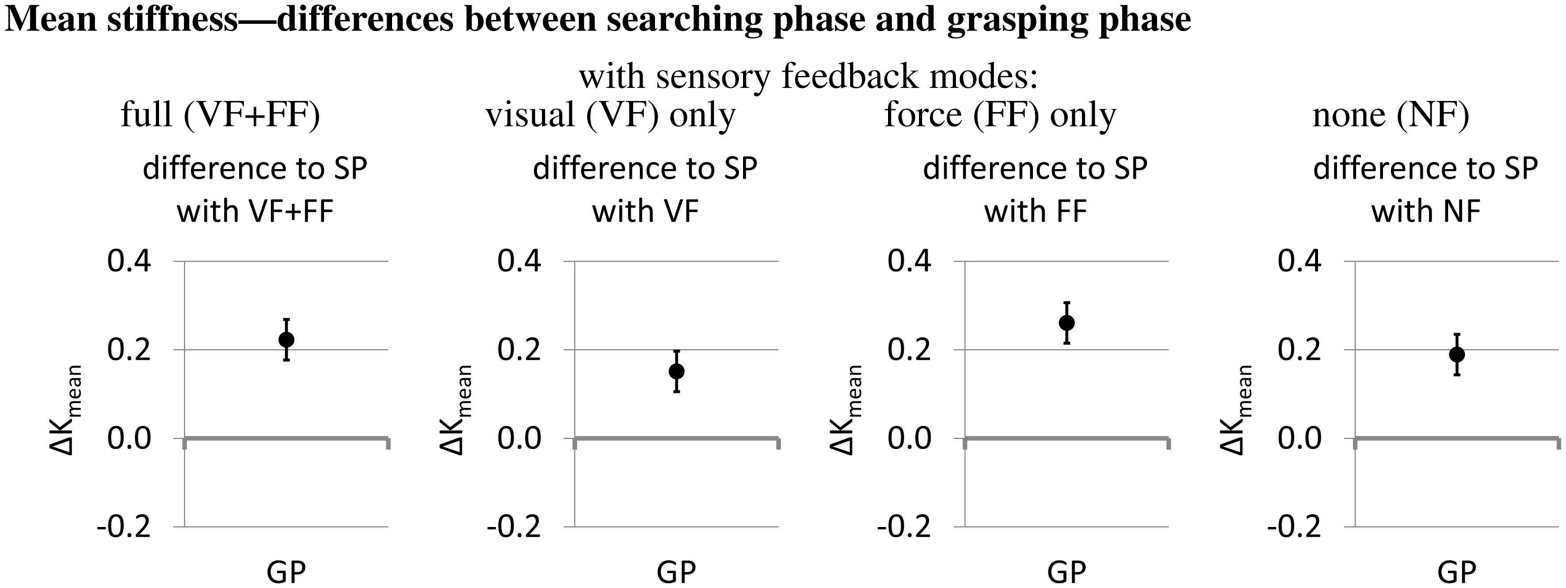
Differences between searching phase (SP) and grasping phase (GP) (estimates and 95% confidence intervals from fitting the mixed model). The searching phase is the baseline.

To answer the question how the operators adjust the stiffness in the variable-stiffness mode during the course of the trial, it is interesting how the stiffness changes between the searching phase and the grasping phase and how it is affected by the sensory feedback mode. Figure 11 compares the grasping phase to the baseline of the searching phase. In the grasping phase, the mean normalized stiffness is about 0.2 units higher than in the searching phase. This difference between searching phase and grasping phase occurs during all sensory feedback conditions.

**Figure 12:**
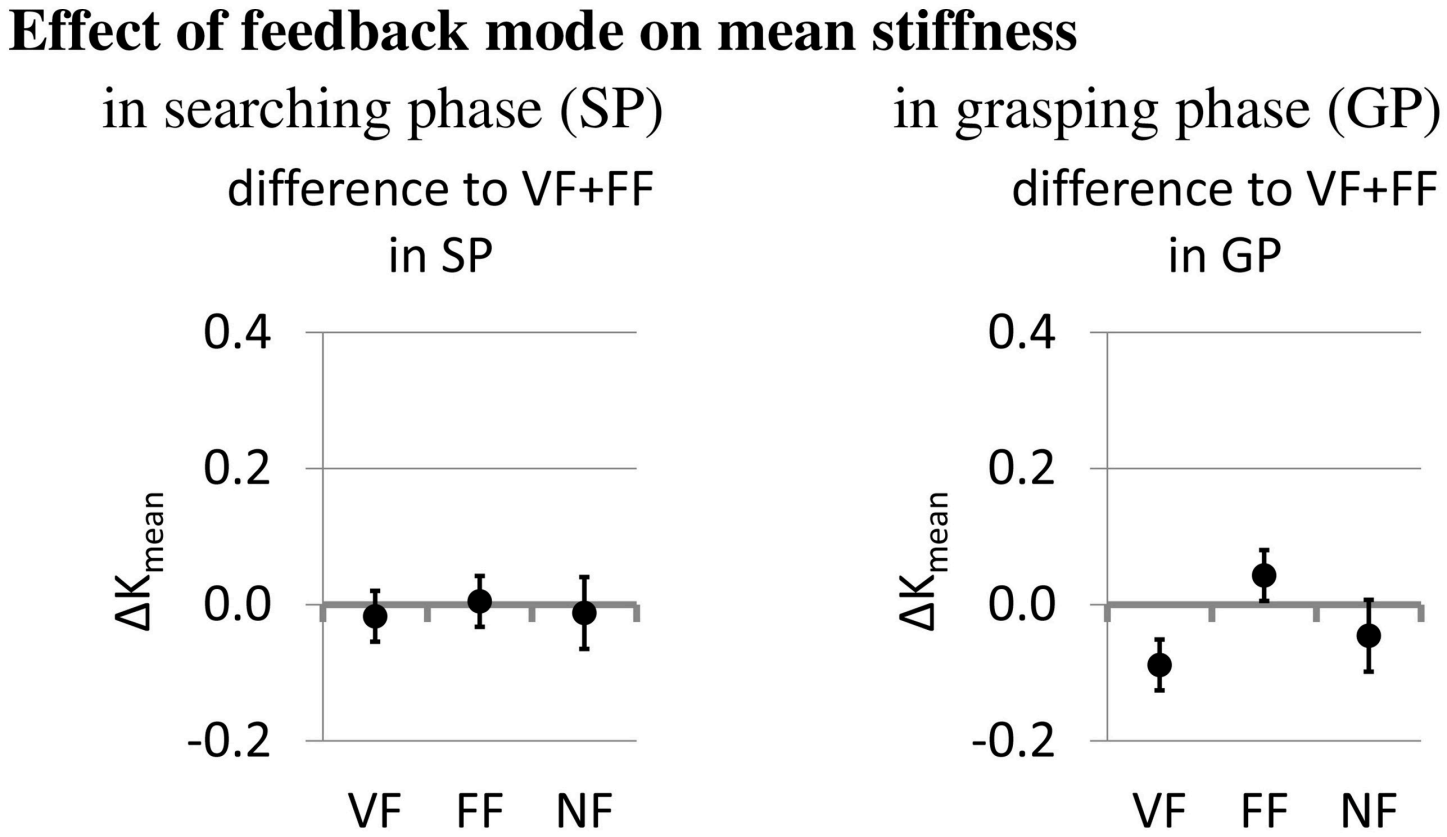
The effect of the feedback modes on the mean stiffness (estimates and 95% confidence intervals from fitting the mixed model).

The effect of sensory feedback itself on the mean normalized stiffness is shown in Figure 12. In the grasping phase (diagram on the right), lack of force feedback leads to a decrease in mean normalized stiffness by about 0.08 units (VF), lack of visual feedback to an increase by about 0.04 units (FF), and lack of both feedback modalities to a decrease by about 0.04 units (NF). While not evident from the mean stiffness data, subjects reported to increase grip stiffness in the searching phase under conditions of sensory impairment in order to generate higher contact forces which could be felt at the lower arm via the splint. For exemplary time series of stiffness tuning and feedback force under different sensory conditions please see the Appendix.

### 3.3. Observations of strategies for exploiting environmental constraints

In the questionnaire, five out of six operators reported using the environment to get a secure grasp on the object.

**Figure 13:**
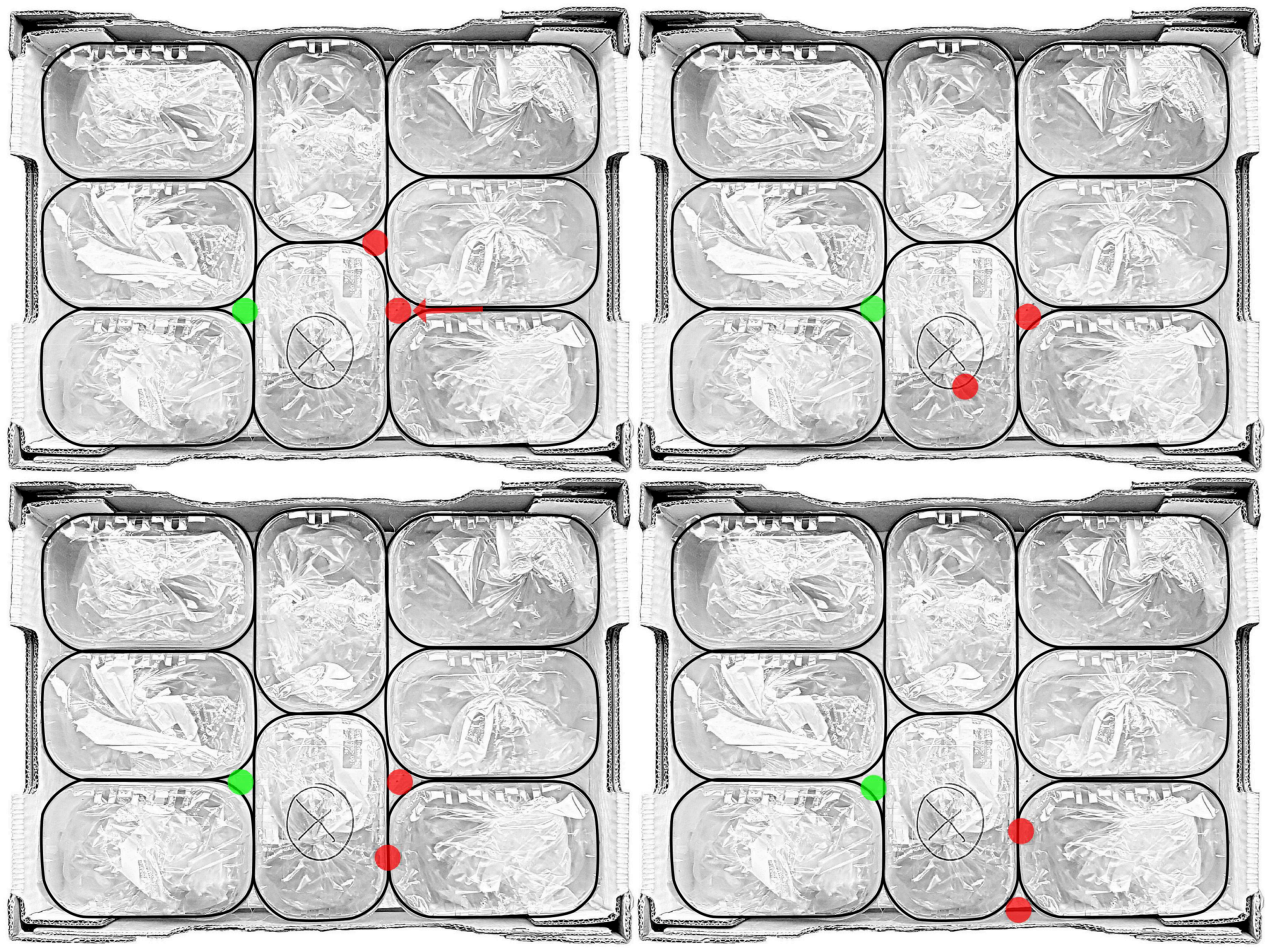
Overview of used strategies to exploit the environment in order to successfully grasp the object: **(Upper left)** motion along the crack between two boxes (red arrow) before placing the WHISG fingers (red dots) and thumb (green dot), (**Upper right**) two finger grasp, (**Lower left / right**) three finger grasps with higher contact forces.

Figure 13 shows a comparison of different strategies that were used to grasp the object. Red dots depict the placement of the WHISG fingers whereas the green dot is the placement of the WHISG thumb. All strategies show a distinct exploitation of the environment: in the upper left, one finger was moved along the crack between two boxes to find the free space. By rotating the operator's arm and therefore the WHISG hand, the second finger was placed in the space above, before the thumb was placed in the space opposite of the fingers, ensuring a secure three-fingered grasp. However, in 94% of all grasp trials, operators placed one finger on top of the box and fixed the object between thumb and one finger (see Figure 13 upper right). In most trials, operators reported to have trouble seeing the lower of the two fingers. The bottom two strategies show three finger grasps with higher contact forces because either the upper or lower WHISG finger had to be placed in the crack between two boxes which required more force than placing the fingers in the free spaces. This sometimes led to squashing the lid of the box and therefore potential damage to the object content.

## 4. Discussion and conclusions

In this study, picking and placing of small-fruit containers from a tightly packed set by the variable-stiffness WHISG robotic hand was investigated under different stiffness modes and different levels of sensory feedback. The hand was positioned and controlled by human operators as proxies for a robotic arm and a control computer. It was shown that the WHISG hand is suitable for consistently performing the task well within the time constraint of 2 min even when the controller received only a limited amount of sensory feedback. However, it was also shown that the task could even be completed by a mechanically simpler hand with a fixed stiffness behavior.

Regarding the influence of sensory feedback on the grasping performance, it was shown that high-quality visual feedback tends to decrease task completion time, number of grasping attempts, and gripping torques. The influence of force feedback was less decisive, with a tendency of increasing task completion time, number of grasping attempts, and gripping torques when using a low-stiffness hand in the absence of visual feedback, but slightly decreasing gripping torques otherwise. This matches the result of Laghi et al. (2017) that visual feedback plays a more significant role in contact recognition than force feedback. Regarding the further result of Laghi et al. (2017) that the presence of force feedback decreases the influence of visual feedback, our results show a confirming tendency under some experimental conditions and a contradicting tendency under other experimental conditions.

Despite the lack of clear benefit on the measured grasping performance, the majority of operators reported in the questionnaire that they found force feedback helpful. This is similar to the findings of Godfrey et al. (2013), where subjects reported lower physical and mental effort when vibro-tactile feedback was present.

The hypothesis that variable stiffness can better compensate lack of sensory feedback than fixed stiffness could not be confirmed comprehensively. Under conditions of impaired visual feedback, high fixed stiffness tended to provide the lowest task completion times and least number of grasping attempts. When high-quality visual feedback was given, low fixed stiffness tended to perform at similar speed and number of attempts as the high-stiffness and variable-stiffness hands, but tended to apply somewhat lower gripping forces, which may be beneficial for the handling of delicate goods. Variable stiffness tended to provide a compromise between high and low stiffness when both visual and force feedback were lacking. While under these conditions it performed similarly fast and with a similar number of grasping attempts as high fixed stiffness and faster and with less attempts than low fixed stiffness, it tended to apply somewhat lower gripping torques than high fixed stiffness but higher gripping torques than low fixed stiffness.

The grasp success rate was 100% under all stiffness and feedback modes, which we found surprising. Apart from proving the suitability of the WHISG hand for the given task, this also shows that a more difficult task might have been needed for better discovering possible benefits of variable stiffness in situations of sensory deprivation. In future studies, the task could be made more difficult by tightening the time constraint, by grasping more delicate objects without hurting them and by positioning the robotic hand by a robotic arm to eliminate the inadvertent force feedback on the operator's forearm via the splint.

The investigation of stiffness variation strategies showed that operators apply higher stiffness in the grasping phase than in the searching phase and somewhat higher stiffness in the presence of force feedback than in its absence. The higher stiffness in the grasping phase corresponds to the presumption that the fingers need to withstand the weight of the object during lifting. The higher stiffness in the presence of force feedback could be explained by the fact that the stiffness of the robotic hand is not only a function of the robotic grasping force, but also of the sEMG-controlled FAS pretension level, which is in turn influenced by the contraction and cocontraction of the operator's muscles. In the absence of force feedback, no forces are applied to the operator's fingers, hence the operator can only modulate the stiffness by cocontraction. When force feedback is turned on, the operator additionally contracts to counteract the forces on the finger, which further increases his grip stiffness and the sEMG signals. This is actually more an artifact of the teleimpedance setup than an insight into an optimal stiffness modulation strategy.

The observation of environmental constraint strategies yielded four distinct grasping patterns, one of which enjoyed a striking preference by being used in 94% of the trials. Interestingly, this pattern, which exploits gaps between the containers near their corners, places only two fingers in grasping positions, as opposed to the other patterns, which place three fingers and exploit the cracks between the containers in addition to the gaps. One possible explanation lies in the fact that subjects had difficulties to see the second finger that often ended up unused. A further possible explanation is that forcing the fingers in the crack between the containers required more force and was therefore harder to realize than the placement in the corner gaps.

In conclusion, the study showed that the VSA WHISG hand is suitable for the task of picking and placing small fruit containers from a tightly packed set but that the task may also be done with a simpler SEA hand with fixed stiffness. Furthermore, it showed operators' strategies of exploiting the cracks and gaps between containers to guide compliant fingers to relevant grasp contact points, which may also be useful for autonomously operated robots.

## Author contributions

MH contributed to literature review, implementation of electronics, calibration methods, experiments, data analysis and paper writing. WF developed the robotic hand, supervised the project overall, and contributed in proof reading. GS contributed to the interpretation of data, writing, and proof reading the manuscript. HH developed the idea, supervised the project overall and contributed in data analysis, writing, and proof reading.

### Conflict of interest statement

The authors declare that the research was conducted in the absence of any commercial or financial relationships that could be construed as a potential conflict of interest.

## Supplementary material

Supplementary Figure 1 Grasping action with VF but without FF; black line: raw sEMG signals for the first and second dorsal interossei muscle (MID1, MID2), blue line: commanded stiffness, red dashed line: GFM position, green dashed line: normalized feedback force, black dashed line: maximum WHISG stiffness.

Supplementary Figure 2 Grasping action with VF and FF; black line: raw sEMG signals for the first and second dorsal interossei muscle (MID1, MID2), blue line: commanded stiffness, red dashed line: GFM position, green dashed line: normalized feedback force, black dashed line: maximum WHISG stiffness.

Supplementary Figure 3 Grasping action without VF and FF; black line: raw sEMG signals for the first and second dorsal interossei muscle (MID1, MID2), blue line: commanded stiffness, red dashed line: GFM position, green dashed line: normalized feedback force, black dashed line: maximum WHISG stiffness.

Supplementary Figure 4 Grasping action without VF but with FF; black line: raw sEMG signals for the first and second dorsal interossei muscle (MID1, MID2), blue line: commanded stiffness, red dashed line: GFM position, green dashed line: normalized feedback force, black dashed line: maximum WHISG stiffness.

## Abbreviations

DIP: distal interphalangeal (joint)
DoF: degrees of freedom
DLR: Deutsches Zentrum für Luft- und Raumfahrt, German Aerospace Center
EC: environmental constraints
FAS: Flexible Antagonistic Spring (VSA mechanism)
FF: force feedback
GFM: Grip Force Master (grip position control and force feedback device)
GP: grasping phase
HS: high stiffness
LS: low stiffness
MANOVA: multivariate analysis of variance
MCP: metacarpophalangeal (joint)
MVC: maximum voluntary contraction
PIP: interphalangeal (joint)
SEA: series-elastic actuator
sEMG: surface electromyography
SOMA: Soft-bodied Intelligence for Manipulation (EU project)
SP: searching phase
VF: visual feedback
VF+FF: full feedback (visual and force feedback)
VS: variable stiffness
VSA: variable-stiffness actuator
WHISG: Wearable Hand to Investigate Stiffness in Grasping (robotic hand).

## Appendix

### A1 stiffness tuning process

The necessary time to tune the stiffness is dependent on two factors: the sampling rate of the finger stiffness of the WHISG hand (100 Hz) and the small latency of the motors to get in the commanded position. Overall it takes less than 150 ms to go from the lowest to the highest stiffness setting, assuming that the muscular activity is at or above the determined MVC level (see e.g., Supplementary Figure 2 at around 5.8 s). The stiffness tuning process is independent of the handled object and is only dependent on the muscular activity of the human operator. Assuming the hand of the operator is not perturbed, external disturbances don't affect the finger stiffness of the WHISG hand.

### A2 stiffness tuning and feedback force under different sensory conditions

The following figures show four trials with sEMG-based variable stiffness and different sensory conditions, all performed by operator 5. In all figures, raw sEMG signals for both electrodes are shown in black. MID1 denotes the activity of the first dorsal interossei muscle, MID2 of the second dorsal interossei muscle. Superimposed in red dashed lines is the commanded position of the GFM, in green dashed lines the feedback force normalized to the maximum feedback force over all trials of the operator (both lines percentaged to each particular scale). The black dashed line shows the maximum finger stiffness of the DLR WHISG hand.

Supplementary Figure 1 depicts a trial with VF but without FF available to the operator. The first 2.75 s are spent searching for the open space between the boxes, before the grasp is finally initiated. Due to the VF being intact, the delayed feedback force doesn't matter for the grasping action. With FF disabled, the operator almost used the full range of the GFM which in return, with the high stiffness, resulted in a big feedback force.

In Supplementary Figure 2, both VF and FF was available. In total, two grasping actions were needed for a successful grasp of the object. After the first failed attempt, the operator voluntarily increased finger stiffness and was able to grasp the object—despite the lowered GFM position in comparison to the first attempt. The feedback force is considerably lower than in Supplementary Figure 1 despite the commanded stiffness being equally high which is a result from the lowered GFM position.

With both VF and FF unavailable to the operator (see Supplementary Figure 3), the operator required the full range of the GFM to grasp the object. The low commanded stiffness could be a result of no sensory information available, which would protect the robotic hand in case of unforeseen impacts with the environment. Feedback force is low due to the lower stiffness despite the increased GFM position.

In Supplementary Figure 4, the operator increased the stiffness before initiating the grasp. With the increased feedback force, it was possible to detect the open space even without the visual information. Due to the reduced GFM position, feedback force overall is lowered even though the commanded stiffness is high.

### A3 video

The video attached to this submission exemplary shows the difference between the low and high stiffness setting of the WHISG hand as well as the inherent compliance when handling soft objects, e.g. fruits. Additionally the experimental procedure with and without visual feedback is presented.
