# Supplementary figures and images for: Human-Robotic Variable-Stiffness Grasps of Small-Fruit Containers Are Successful Even Under Severely Impaired Sensory Feedback
Source: Front Neurorobot. 2018 Oct 31;12:70. doi: 10.3389/fnbot.2018.00070 (PMC6220053; doi:10.3389/fnbot.2018.00070)

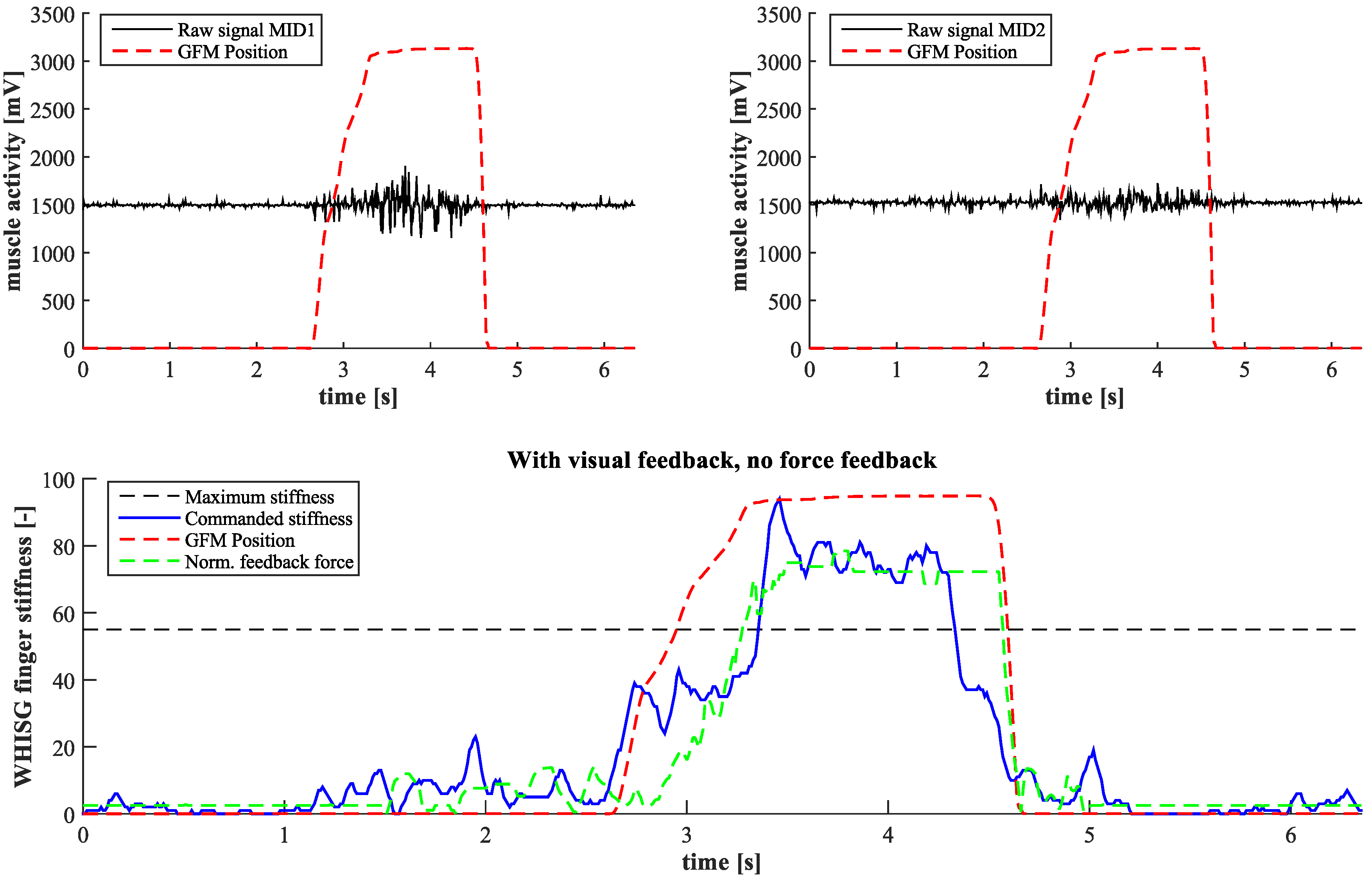

Supplement: Supplementary Figure 1 — Grasping action with VF but without FF; black line: raw sEMG signals for the first and second dorsal interossei muscle (MID1, MID2), blue line: commanded stiffness, red dashed line: GFM position, green dashed line: normalized feedback force, black dashed line: maximum WHISG stiffness. [file Image_1.TIFF]

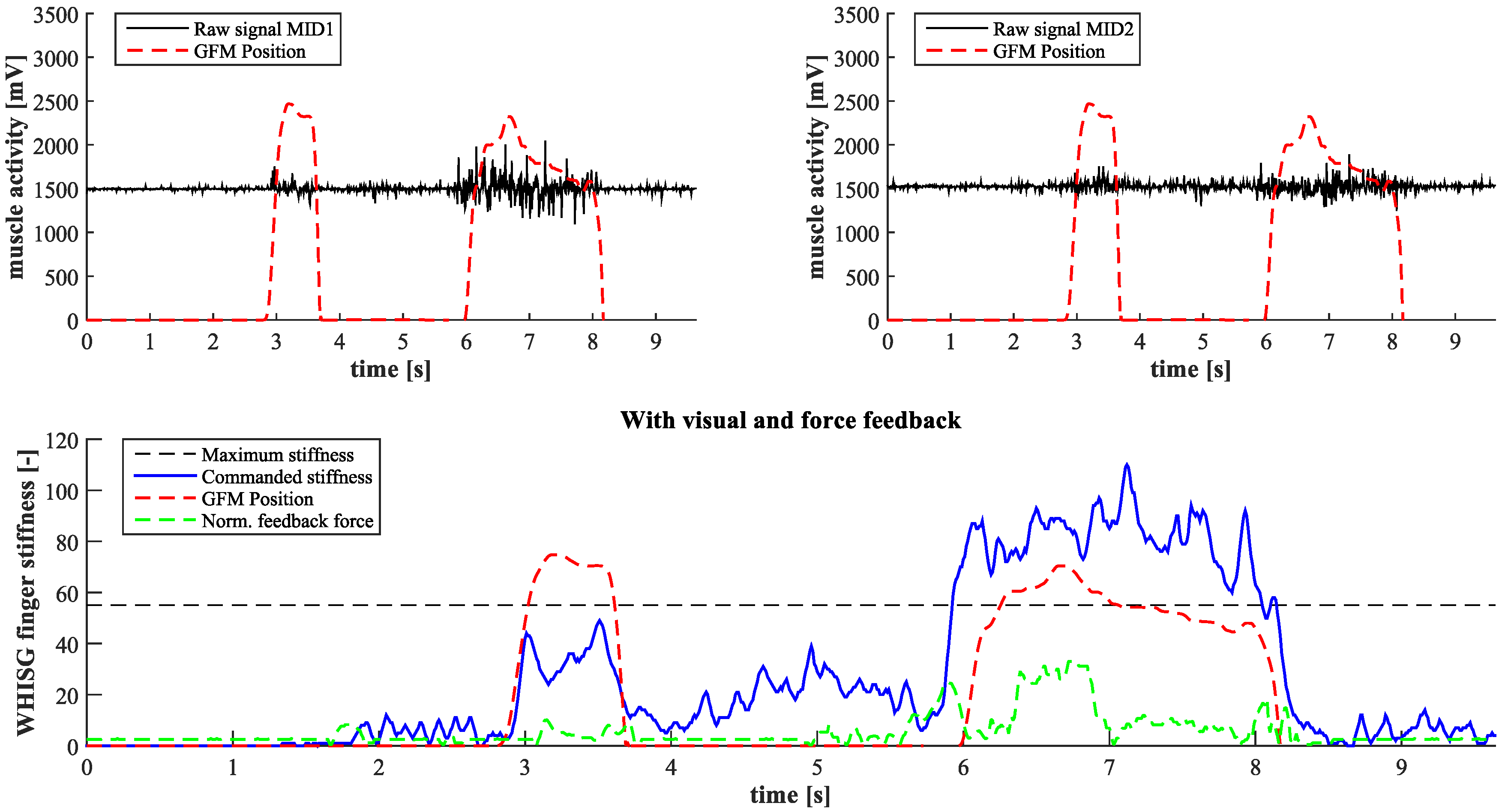

Supplement: Supplementary Figure 2 — Grasping action with VF and FF; black line: raw sEMG signals for the first and second dorsal interossei muscle (MID1, MID2), blue line: commanded stiffness, red dashed line: GFM position, green dashed line: normalized feedback force, black dashed line: maximum WHISG stiffness. [file Image_2.TIFF]

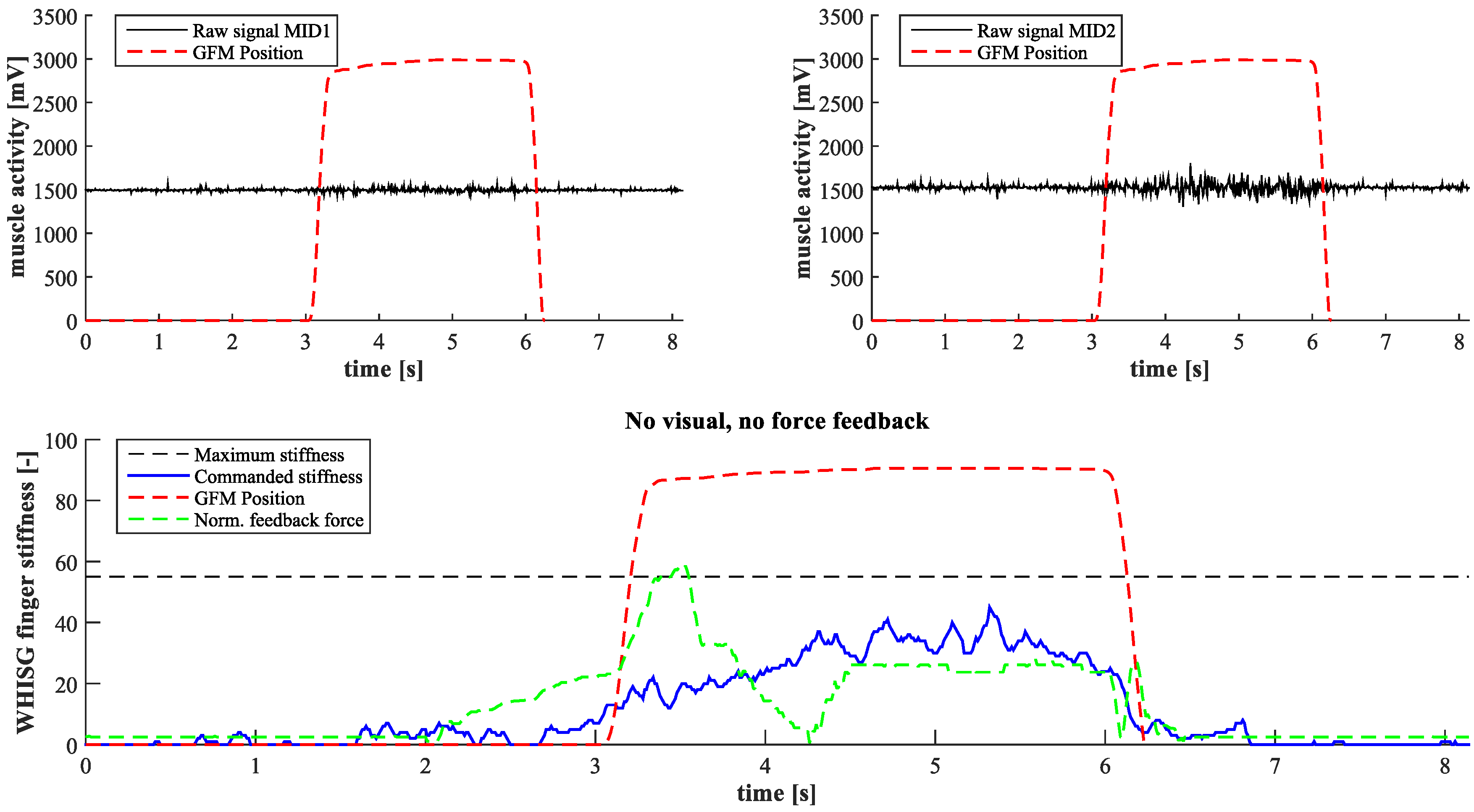

Supplement: Supplementary Figure 3 — Grasping action without VF and FF; black line: raw sEMG signals for the first and second dorsal interossei muscle (MID1, MID2), blue line: commanded stiffness, red dashed line: GFM position, green dashed line: normalized feedback force, black dashed line: maximum WHISG stiffness. [file Image_3.TIFF]

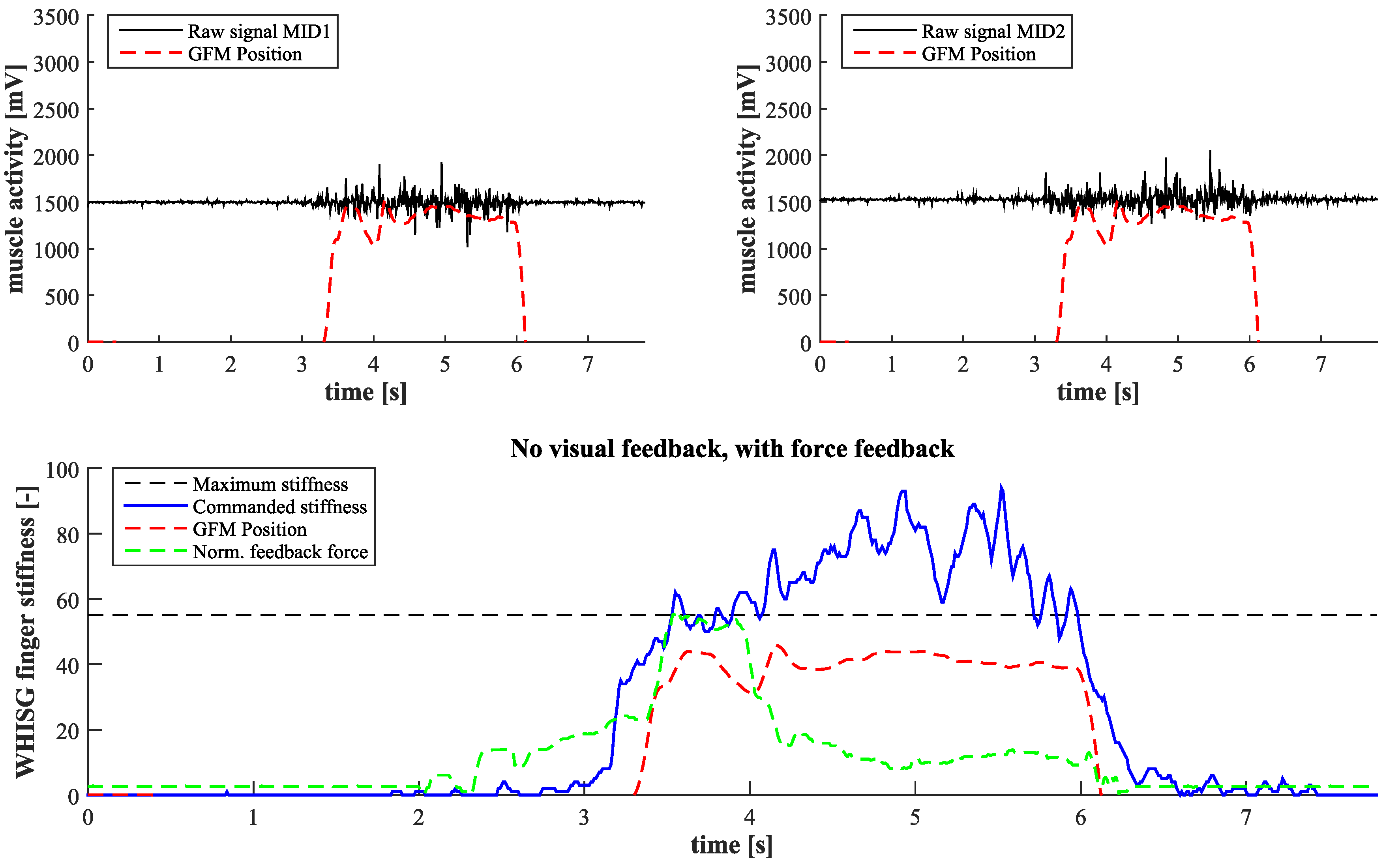

Supplement: Supplementary Figure 4 — Grasping action without VF but with FF; black line: raw sEMG signals for the first and second dorsal interossei muscle (MID1, MID2), blue line: commanded stiffness, red dashed line: GFM position, green dashed line: normalized feedback force, black dashed line: maximum WHISG stiffness. [file Image_4.TIFF]
